# Advantage of Scheduled Upfront Lenvatinib Administration Followed by Transarterial Chemoembolization Therapy Over Lenvatinib Monotherapy in Patients With Unresectable Intermediate‐Stage Hepatocellular Carcinoma: A Multicenter Cohort Study

**DOI:** 10.1002/cam4.71503

**Published:** 2026-01-16

**Authors:** Nobuhito Taniki, Keisuke Ojiro, Ryosuke Kasuga, Yukie Nakadai, Takaya Tabuchi, Po‐Sung Chu, Shingo Usui, Hitomi Hoshi, Fumihiko Kaneko, Akihiro Yamaguchi, Jun Koizumi, Hirotoshi Ebinuma, Masashi Tamura, Jitsuro Tsukada, Masanori Inoue, Seishi Nakatsuka, Yasushi Hasegawa, Yuta Abe, Minoru Kitago, Masahiro Jinzaki, Yuko Kitagawa, Takanori Kanai, Nobuhiro Nakamoto

**Affiliations:** ^1^ Division of Gastroenterology and Hepatology, Department of Internal Medicine Keio University School of Medicine Tokyo Japan; ^2^ Department of Gastroenterology Ichikawa General Hospital, Tokyo Dental College Chiba Japan; ^3^ Department of Gastroenterology and Hepatology Saitama City Hospital Saitama Japan; ^4^ Division of Gastroenterology, Department of Internal Medicine National Hospital Organization Saitama National Hospital Saitama Japan; ^5^ Department of Diagnostic Radiology and Radiation Oncology, School of Medicine Chiba University Chiba Japan; ^6^ Department of Gastroenterology, School of Medicine International University of Health and Welfare Chiba Japan; ^7^ Department of Radiology Keio University School of Medicine Tokyo Japan; ^8^ Department of Radiology Fujita Health University School of Medicine Toyoake Japan; ^9^ Department of Surgery Keio University School of Medicine Tokyo Japan

**Keywords:** hepatocellular carcinoma, lenvatinib, transarterial chemoembolization

## Abstract

**Background and Aim:**

Combining systemic chemotherapy with transarterial chemoembolization (TACE) has demonstrated improved outcomes, with promising results for the efficacy of lenvatinib pretreatment combined with TACE in single‐arm studies involving patients with hepatocellular carcinoma (HCC). This study aimed to evaluate the efficacy of a scheduled upfront lenvatinib combined with a TACE regimen in patients with HCC, representing both the first study to comparatively analyze this approach and to focus specifically on patients unsuitable for TACE.

**Methods:**

We conducted a multicenter retrospective study between 2018 and 2024, enrolling 41 patients with unresectable Barcelona Clinic Liver Cancer intermediate‐stage HCC who were considered unsuitable for TACE owing to factors such as exceeding the up‐to‐7 criteria, having infiltrative HCC, or multiple asynchronous recurrent HCC. Of these patients, 25 received upfront lenvatinib administration prior to TACE (LEN‐TACE group), followed by continuous lenvatinib and on‐demand TACE, and 16 received lenvatinib monotherapy.

**Results:**

Radiological evaluation revealed significantly higher complete response (CR) and objective response rates (ORR) in the LEN‐TACE group than in the lenvatinib group. The median overall survival (OS) was not reached in the LEN‐TACE group, whereas it was 16.2 months in the lenvatinib monotherapy group, indicating a significantly superior OS in the LEN‐TACE group (hazard ratio [HR]: 2.99; 95% CI: 1.01–8.95; *p* = 0.0496). Superiority in both PFS and OS was also observed in the propensity score‐matched (PSM) cohort for the LEN‐TACE group.

**Conclusion:**

Scheduled upfront lenvatinib combined with TACE is superior to lenvatinib monotherapy for intermediate‐stage HCC, particularly in patients unsuitable for TACE.

**Abbreviations**
95% CIs: 95% confidence intervals
AFP: alpha‐fetoprotein
ALBI score: albumin‐bilirubin score
BCLC: The Barcelona Clinic Liver Cancer prognosis and treatment strategy
BMI: body mass index
CR: complete response
DCP: des‐γ‐carboxy prothrombin
DCR: disease control rate
FGF: fibroblast growth factor
HCC: hepatocellular carcinoma
HR: hazard ratio
LEN: Lenvatinib
mRECIST: modified Response Evaluation Criteria in Solid Tumors
NE: not evaluable
ORR: objective response rate
OS: overall survival
PD: progressive disease
PFS: progression free survival
PR: partial response
RECICL: Response Evaluation Criteria in the Cancer of Liver
RECIST: Response Evaluation Criteria in Solid Tumors
SD: stable disease
TACE: transarterial chemoembolization
VEGF: vascular endothelial growth factor

## 1 Introduction

Hepatocellular carcinoma (HCC) is the third‐leading cause of cancer‐related deaths in 46 countries [1]. Barcelona Clinic Liver Cancer (BCLC) intermediate‐stage HCC encompasses a highly heterogeneous patient population, necessitating tailored treatment decisions to the specific clinical circumstances of each individual [2, 3]. Previous studies have identified various factors associated with resistance to transarterial chemoembolization (TACE) therapy. The up‐to‐7 criteria, where seven represents the combined total of the largest tumor diameter (in centimeters) and the number of tumors, have been used to subclassify patients and predict TACE refractoriness [4, 5, 6]. Radiological characteristics are also associated with prognosis after TACE [7]. HCCs with unencapsulated or infiltrative radiological features are associated with a poorer prognosis [8]. Additionally, we previously reported that multiple asynchronous recurrences are predictive factors of refractoriness to surgical and locoregional therapies [9]. Such clinical situations where patients are unsuitable for TACE can be managed with systemic chemotherapy. Lenvatinib, a molecular‐targeted agent that inhibits key receptors involved in tumor angiogenesis—including the vascular endothelial growth factor (VEGF) and fibroblast growth factor (FGF) receptors—has proven to be an effective treatment for such patients [10]. Furthermore, combining molecular‐targeted agents with VEGF inhibitors and TACE can enhance therapeutic efficacy [11]. The efficacy of lenvatinib‐TACE sequential therapy, where lenvatinib and TACE are alternated during treatment periods, has also been reported [12, 13, 14, 15]. Notably, combining lenvatinib with TACE, where lenvatinib is administered prior to TACE, enhances the therapeutic efficacy of TACE. This approach, followed by continuous lenvatinib and on‐demand TACE, has demonstrated promising results in single‐arm trials [16, 17, 18]. However, evidence from comparative studies evaluating these approaches is currently lacking. Furthermore, as the existing single‐arm studies included patients with BCLC stages A to C, further investigation is necessary to assess the efficacy of this approach in patients with BCLC‐B who are considered unsuitable for TACE, particularly those for whom TACE alone is anticipated to result in suboptimal outcomes.

In this study, we aimed to investigate the effectiveness of scheduled lenvatinib pre‐administration before TACE, followed by continuous lenvatinib and on‐demand TACE, in patients with BCLC‐B. We focused on patients considered unsuitable for TACE, such as those exceeding the up‐to‐7 criteria, with infiltrative HCC, or multiple asynchronous recurrent HCC.

## 2 Methods

### 2.1 Patients

This retrospective observational study was conducted within a multicenter cohort in Japan between June 2018 and February 2024. Patients were recruited from four liver centers across Japan (Keio University Hospital, Tokyo Dental College Ichikawa General Hospital, Saitama City Hospital and National Hospital Organization Saitama Hospital). The inclusion criterion was unresectable BCLC intermediate‐stage HCC unsuitable for surgical resection or thermal ablation therapy, particularly in cases where TACE was not feasible. This encompassed patients with tumors exceeding the up‐to‐7 criteria, infiltrative HCC, or more than two episodes of asynchronous recurrent HCC. The exclusion criteria included 20 or more tumors and an ECOG performance status of 2–4 (Figure S1). All consecutive patients with BCLC‐B stage HCC who received lenvatinib during the study period were identified. A total of 47 patients with BCLC‐B stage HCC treated with lenvatinib were screened, and ultimately, 41 patients were included in the study. The key imaging features of HCC, as outlined by the American Association for the Study of Liver Diseases criteria and the Liver Imaging Reporting and Data System (LI‐RADS), include arterial phase hyperenhancement, lesion size, washout appearance, enhancing capsule appearance, and threshold growth observed on multiphase contrast‐enhanced CT or MRI [19, 20].

### 2.2 Medical Care

The patients were treated using two approaches: the first group received a scheduled upfront lenvatinib prior to TACE, followed by continuous lenvatinib and on‐demand TACE (LEN‐TACE group), while the second group received lenvatinib monotherapy (LEN monotherapy group). The decision to administer LEN‐TACE or proceed with LEN monotherapy was made at the discretion of each facility. The treatment protocol is outlined in Figure S1.

In the LEN‐TACE group, lenvatinib was administered for 14–21 days before the first TACE, paused 2 days prior to the procedure, and resumed after the resolution of post‐TACE syndrome (e.g., fever, elevated AST/ALT levels, or abdominal pain), as determined by the investigator. Patients continued lenvatinib treatment until disease progression, unacceptable toxicity, initiation of conversion therapy, or withdrawal of consent. Continuation of lenvatinib treatment after disease progression was permitted based on the discretion of each facility.

### 2.3 Lenvatinib Administration Protocol and TACE Procedure

Lenvatinib was administered orally once daily at a dose of 12 mg for patients weighing 60 kg or more, and 8 mg for those weighing < 60 kg. Lenvatinib was either reduced or temporarily halted in patients who experienced grade ≥ 3 severe adverse events (AEs) or any unacceptable grade 2 drug‐related AEs. AEs were evaluated according to the National Cancer Institute Common Terminology Criteria for Adverse Events (version 5.0). Dose adjustments were made at the discretion of each facility. Subsequent TACE sessions were performed on demand as needed. Conventional TACE involved the intra‐arterial administration of lipiodol combined with either epirubicin or miriplatin, followed by the injection of an embolic agent (Gelpart) to interrupt blood flow. Drug‐eluting bead transarterial chemoembolization was performed selectively or super‐selectively using 100–300 μm DC‐Beads (Eisai) loaded with epirubicin at a concentration of 50 mg/2 mL of beads.

### 2.4 Outcome Assessment

Treatment response was evaluated using dynamic CT or MRI, according to the Response Evaluation Criteria in Solid Tumors (RECIST) v1.1, modified RECIST (mRECIST) criteria, and Response Evaluation Criteria in the Cancer of Liver (RECICL) [21, 22]. Tumors were assessed once within the first 8 weeks and every 8–16 weeks thereafter. Patient characteristics, death or disease progression, and therapeutic responses were analyzed retrospectively. Overall survival (OS) was defined as the time from lenvatinib treatment initiation to death from any cause, with the censoring date for surviving patients being the last observation date. Progression‐free survival (PFS) was defined as the time from the initiation of lenvatinib treatment to radiological progression, assessed using the mRECIST criteria for the LEN monotherapy group and the RECICL criteria for the LEN‐TACE group, or death from any cause. For patients without disease progression or death, the censoring date was the date of the last radiological assessment. The area of lipiodol retention in the nodule more than 1 month after TACE was considered necrotic tissue [23]. Radiological imaging evaluations were reviewed retrospectively by at least two independent investigators.

### 2.5 Statistical Analysis

Data analysis was conducted using SPSS version 28 (IBM Corp., Armonk, NY, USA), with results presented as medians with interquartile ranges or mean ± standard deviations, depending on the context. Graphical representations were created using Prism 9.0 (GraphPad Software Inc., San Diego, CA, USA). Group differences were evaluated using the Student's *t*‐test for continuous variables, while categorical variables were analyzed with the chi‐squared test. Hazard ratios (HRs) and 95% confidence intervals (CIs) were calculated through Cox proportional hazards models. Kaplan–Meier survival curves were generated, and median survival times were determined. A *p*‐value of < 0.05 was deemed statistically significant across all analyses.

### 2.6 Propensity Score Matching (PSM) Analysis

To minimize selection bias and enable a balanced comparison between patients treated with LEN‐TACE and those receiving LEN monotherapy, we employed PSM at a 1:1 ratio. This method aimed to establish matched groups for a more robust survival analysis.

The propensity scores were generated using a logistic regression model incorporating key variables, including body mass index (BMI), albumin‐bilirubin (ALBI) score, the largest tumor diameter, and the number of tumors. The goodness‐of‐fit for the model was assessed using C‐statistics. Matching was performed with a caliper width of < 0.2 times the pooled standard deviation of the propensity scores, ensuring a high degree of comparability between the two groups.

## 3 Results

### 3.1 Patient Characteristics

**FIGURE 1:**
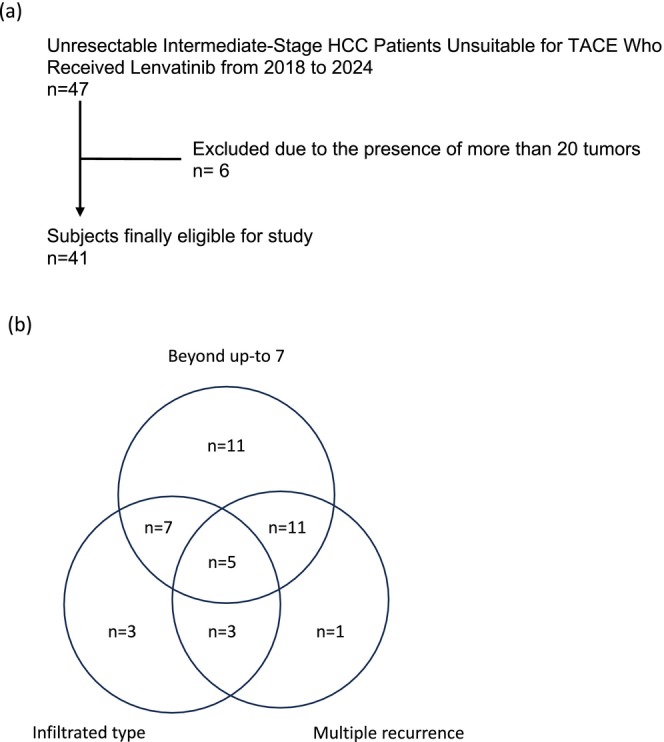
Details of the eligible patients. (a) Flow chart of eligible patients. (b) Venn diagram showing the distribution of patients fulfilling criteria for unsutability for TACE. HCC, hepatocellular carcinoma; TACE, trans‐arterial chemoembolization.

After registration, 41 patients met the inclusion criteria, all of whom had conditions that rendered them unsuitable for TACE. Of these, 34 patients had numerous large tumors exceeding the up‐to‐7 criteria, 18 had infiltrative‐type HCC, and 20 had multiple asynchronous recurrence episodes (Figure 1). A total of 26 patients (63%) were treated using the LEN‐TACE approach, while the remaining 15 patients (37%) received LEN monotherapy. Table 1 presents the characteristics of the two groups, revealing that body mass index (BMI) was the only significant difference in the unmatched cohort. There were no significant differences between the two groups in other factors, including age, sex, liver disease etiology, tumor characteristics (such as the number of tumors and maximum diameter), hepatic reserve assessed using the Child–Pugh and ALBI scores, tumor markers, proportion of infiltrative tumors in radiological images, history of asynchronous recurrences, or other systemic chemotherapies. Following PSM, there were no statistically significant differences in patient characteristics, including factors related to tumor condition and hepatic reserve, between the two groups.

**TABLE 1:** Baseline demographic and clinical characteristics of patients enrolled in this study.

Variable	Unmatched cohort			PSM cohort		
	LEN‐TACE (*n* = 25)	LEN monotherapy (*n* = 16)	*p*	LEN‐TACE (*n* = 11)	LEN monotherapy (*n* = 11)	*p*
Age (years), median (range)	79 (57–90)	74.5 (48–85)	0.2001	79 (58–88)	78 (48–85)	0.3252
Male, *n* (%)	22 (88)	13 (81)	0.5549	9 (82)	11 (100)	0.1380
BMI (kg/m^2^)	24.5 ± 2.9	22.2 ± 2.1	0.0107*	22.9 ± 2.4	22.6 ± 2.1	0.7474
Hepatitis B, C virus infection, *n* (%)	7 (28)	7 (44)	0.3018	4 (36)	2 (18)	0.3384
Child‐Pugh score	5.5 ± 0.9	5.6 ± 0.6	0.6781	5.6 ± 0.8	5.5 ± 0.5	0.7574
ALBI score	−2.53 ± 0.42	−2.30 ± 0.43	0.0999	−2.37 ± 0.49	−2.44 ± 0.32	0.6919
Number of tumor	7.0 ± 6.0	8.6 ± 4.7	0.3943	8.5 ± 7.1	9.1 ± 5.0	0.8106
Largest tumor diameter (cm)	4.3 ± 3.3	4.5 ± 2.6	0.8208	3.9 ± 3.5	4.1 ± 2.2	0.8864
Within up‐to‐7 criterion, *n* (%)	2 (13)	5 (20)	0.5266	1 (9)	2 (18)	0.5344
AFP (ng/dL)	541 ± 1907	1632 ± 5496	0.3744	884 ± 2757	2367 ± 6588	0.4990
DCP (mAU/mL)	1624 ± 3467	3839 ± 9797	0.3156	2000 ± 4095	4470 ± 11,926	0.5251
Infiltrative type, *n* (%)	10 (40)	8 (50)	0.5294	4 (36)	6 (55)	0.3918
Multiple recurrence, *n* (%)	14 (56)	6 (37.5)	0.2458	6 (55)	4 (36)	0.3918
Previous systemic chemotherapy, *n* (%)	3 (12)	3 (19)	0.5549	2 (18)	3 (27)	0.6109

*Note:* Asterisks indicate statistically significant differences of means (0.01 ≤ *p < 0.05).

Abbreviations: AFP, alpha‐fetoprotein; ALBI score, albumin‐bilirubin score; BMI, body mass index; DCP, des‐γ‐carboxy prothrombin; LEN, lenvatinib; TACE, transarterial chemoembolization.

### 3.2 Efficacy in Tumor Responses of Scheduled Upfront Lenvatinib Combined With TACE Regimen

Table 2 presents the optimal radiological responses. According to RECIST, the LEN‐TACE group exhibited rates of complete response (CR), partial response (PR), stable disease (SD), and progressive disease (PD) of 16%, 52%, 16%, and 16%, respectively. The overall response rate (ORR), which combined the CR and PR, was 68%. Conversely, the LEN monotherapy group exhibited rates of CR, PR, SD, and PD of 0%, 25%, 44%, and 25%, respectively, with an ORR of 25%. According to the mRECIST, the LEN‐TACE group had rates of CR, PR, SD, and PD of 40%, 36%, 12%, and 12%, respectively, with an ORR of 76%. The LEN monotherapy group exhibited rates of CR, PR, SD, and PD of 6%, 31%, 31%, and 25%, respectively, with an ORR of 38%. Statistically significant differences in CR and ORR between the two groups were evaluated using RECIST and mRECIST. Similar results were observed in the PSM cohort; according to RECIST and mRECIST, the LEN‐TACE group had rates of CR of 18% and 55%, and ORR of 82% and 91%, respectively. The LEN monotherapy group exhibited rates of CR of 0% and 0%, and past ORR of 18% and 27%, respectively, indicating significant differences in the CR and ORR between the two groups. The LEN‐TACE group exhibited a notably superior radiological response, particularly in terms of CR and ORR.

**TABLE 2:** Best radiological response.

Best response	Unmatched cohort						PSM cohort					
	RECIST			mRECIST			RECIST			mRECIST		
	LEN‐TACE (*n* = 25)	LEN mono therapy (*n* = 16)	*p*	LEN‐TACE (*n* = 25)	LEN mono therapy (*n* = 16)	*p*	LEN‐TACE (*n* = 11)	LEN mono therapy (*n* = 11)	*p*	LEN‐TACE (*n* = 11)	LEN mono therapy (*n* = 11)	*p*
CR	4 (16)	0 (0)	0.0126*	10 (40)	1 (6)	0.0105*	2 (18)	0 (0)	0.0257*	6 (55)	0 (0)	0.0455*
PR	13 (52)	4 (25)	0.082	9 (36)	5 (31)	0.7537	7 (64)	2 (18)	0.0266*	4 (36)	3 (27)	0.6467
SD	4 (16)	7 (44)	0.0521	3 (12)	5 (31)	0.1335	1 (9)	5 (45)	0.0477*	1 (9)	4 (36)	0.1168
PD	4 (16)	4 (25)	0.4821	3 (12)	4 (25)	0.2864	1 (9)	4 (36)	0.1168	0 (0)	4 (36)	0.0037**
NE	0 (0)	1 (6)		0 (0)	1 (6)		0 (0)	0 (0)		0 (0)	0 (0)	
Response rate												
ORR	17 (68)	4 (25)	0.0063**	19 (76)	6 (38)	0.0133*	9 (82)	2 (18)	0.0019**	10 (91)	3 (27)	0.0014**
DCR	21 (84)	11 (69)	0.2547	22 (88)	11 (69)	0.1335	10 (91)	7 (64)	0.1168	11 (100)	7 (64)	0.0037**

*Note:* Asterisks indicate statistically significant differences of means (0.01 ≤ *p < 0.05; 0.001 ≤ **p < 0.01).

Abbreviations: CR, complete response; DCR, disease control rate; NE, not evaluable; ORR, objective response rate; PD, progressive disease; PR, partial response; SD, stable disease.

### 3.3 Efficacy in Prolonged Prognosis of Scheduled Upfront Lenvatinib Combined With TACE Regimen

**FIGURE 2:**
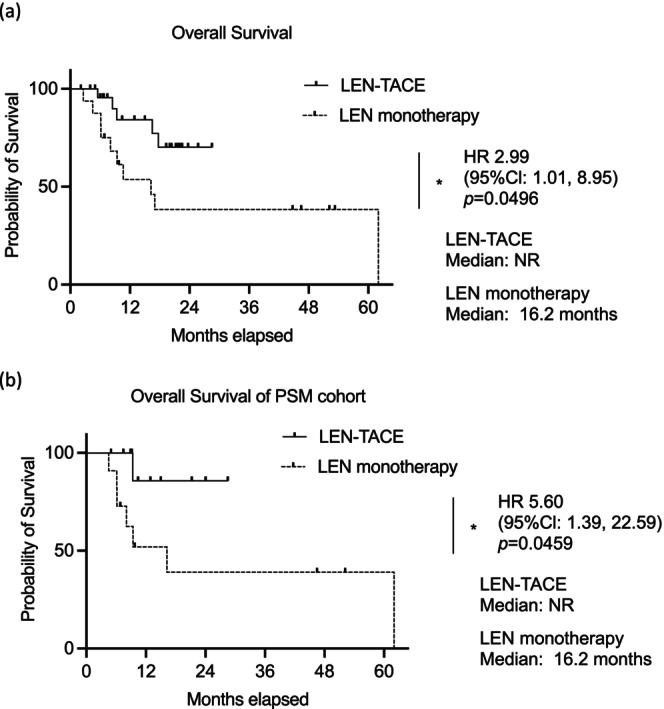
Kaplan–Meier curves of overall survival. Overall Survival of (a) whole cohort. (b) Propensity score‐matched cohort. Asterisks indicate statistically significant differences of means (0.01 ≤ *p < 0.05). 95% CIs, 95% confidence intervals; HR, hazard ratio; LEN, lenvatinib; NR, not reached; TACE, transarterial chemoembolization.

**FIGURE 3:**
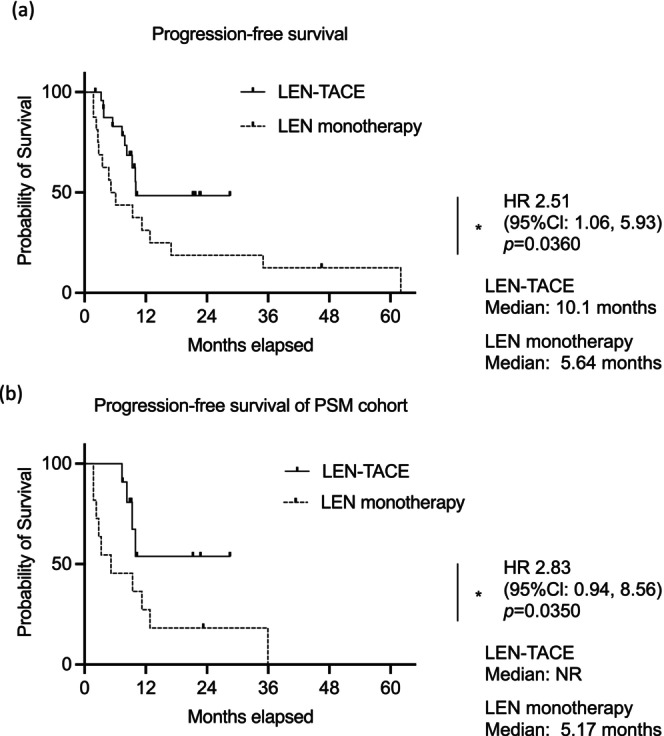
Kaplan–Meier curves of progression‐free survival. Progression‐free survival of (a) whole cohort. (b) Propensity score‐matched cohort. Asterisks indicate statistically significant differences of means (0.01 ≤ *p < 0.05). 95% CIs, 95% confidence intervals; HR, hazard ratio; LEN, lenvatinib; NR, not reached; TACE, transarterial chemoembolization.

The median OS was not reached in the LEN‐TACE group, whereas it was 16.2 months in the LEN monotherapy group, indicating a significantly superior OS in the LEN‐TACE group (hazard ratio [HR], 2.99; 95% CI, 1.01–8.95; *p* = 0.0496). After PSM, the median OS remained unreached in the LEN‐TACE group, whereas it was 16.2 months in the LEN monotherapy group, indicating significantly superior OS in the LEN‐TACE group (HR, 5.60; 95% CI, 1.39–22.59; *p* = 0.0459; Figure 2). The median PFS was 10.1 months in the LEN‐TACE group and 5.6 months in the LEN monotherapy group, demonstrating significantly improved PFS in the LEN‐TACE group (HR, 2.51; 95% CI, 1.06–5.93; *p* = 0.0360). Following PSM, the median PFS remained unreached in the LEN‐TACE group, whereas it was 5.2 months in the LEN monotherapy group (HR, 2.83; 95% CI, 0.94–8.56; *p* = 0.0350; Figure 3). The LEN‐TACE group exhibited a notably superior prognosis and duration of disease control.

## 4 Discussion

Combining molecularly targeted agents with TACE aims to enhance vascular permeability, lower interstitial pressure, and improve drug delivery, thereby increasing the therapeutic effectiveness of TACE [24, 25, 26, 27]. Previous studies have demonstrated that lenvatinib exerts these favorable effects [28, 29]. Moreover, TACE‐induced hypoxia activates HIF‐1α, leading to elevated VEGF levels and promoting tumor growth [30]. The use of molecular‐targeted agents before or after TACE can suppress this VEGF surge, preventing tumor progression. Consequently, basic research has demonstrated the synergistic potential and synergistic effects of lenvatinib and TACE, and the clinical utility of this combination has been established (Table 3). Specifically, Kawamura et al. were the first to report the efficacy of LEN‐TACE sequential therapy, comparing continued LEN‐TACE sequential therapy with alternative treatments used as subsequent therapy for patients diagnosed with PD following TACE or LEN‐TACE sequential therapy [12]. Shimose et al. also reported the efficacy of alternating lenvatinib and transarterial therapy, comparing switching to TACE plus lenvatinib versus continuing lenvatinib after a PD diagnosis during lenvatinib treatment [13]. Ando et al. reported the efficacy of LEN‐TACE sequential therapy in patients who achieved PR or SD following lenvatinib administration [14]. Kuroda et al. observed favorable treatment outcomes using LEN‐TACE sequential therapy in cases where an 8‐week lenvatinib preload proved effective [15]. Peng et al. also reported the superiority of lenvatinib and on‐demand TACE compared with lenvatinib monotherapy [31]. The results of LEN‐TACE sequential therapy and LEN plus on‐demand TACE therapy have led to the development of the LEN‐TACE combination therapy, in which lenvatinib is administered prior to TACE, followed by continuous lenvatinib administration with on‐demand TACE. Kudo et al. were the first to adopt this approach and conducted the TACTICS‐L trial, a phase 2 single‐arm trial, which demonstrated the benefits of a 2–3‐week short‐term pre‐administration of lenvatinib before TACE, followed by continuous lenvatinib and on‐demand TACE [16]. Furthermore, Tachiiri et al. reported that even a short pretreatment period was effective of an even shorter pre‐administration period, with a 4‐day course of lenvatinib before TACE, demonstrating effectiveness in a single‐arm study [18].

**TABLE 3:** Previous studies on lenvatinib and TACE involving sequential and combination therapies.

No	Author	Study	Protocol	No. of patients	BCLC stage	Up to 7 Criteria		Control arm	Evaluation	Outcome	ORR; *n*, (%)	CR; *n*, (%)	PFS median (months)	OS median (months)
						Within	Beyond							
1	Kawamura et al. Liver Cancer 2020	LEN‐TACE sequential therapy	Continued LEN‐TACE sequential therapy versus alternative treatments as subsequent therapy for patients diagnosed as PD following TACE or LEN‐TACE sequential therapy	12	A–C	—	—	Other or none subsequent treatment	mRECIST	PFS, PPS	—	—	—	—
2	Shimose et al. Cancers 2021	Alternating Lenvatinib and Trans‐Arterial Therapy	A comparison of switching to TACE plus lenvatinib versus continuing lenvatinib after a PD diagnosis on lenvatinib	41	B	6 (15%)	35 (85%)	Lenvatinib (non trans‐arterial therapy)	mRECIST	PFS, OS	—	—	13.6 (treatment period)	NR
3	Ando et al. Oncology 2021	LEN‐TACE sequential therapy	Patients were included if PR or SD was achieved following lenvatinib administration. The included patients received either LEN‐TACE sequential therapy or lenvatinib alone	30	B	7 (23%)	23 (77%)	Lenvatinib	mRECIST	ORR, PFS, OS	9 (47.4%)	3 (15.8%)	11.6	NR
4	Kuroda et al. Liver Cancer 2022	LEN‐TACE sequential therapy	Lenvatinib was administered for 8 weeks as a preload, followed by TACE regardless of the response. Favorable treatment outcomes were observed in cases where the 8‐week lenvatinib preload was effective	63	B–C	—	—	Lenvatinib	mRECIST	ORR, PFS, OS	39 (61.9%)	13 (20.6%)	12.2	31.2
5	Kudo et al. Liver Cancer 2023	LEN‐TACE combination therapy	Lenvatinib was scheduled to be administered 14–21 days prior to the first TACE	62	A–C^†^ †PS1	40 (64.5%)	22 (35.5%)	Single arm	RECICL	ORR, PFS, OS	49 (79%)	33 (53.2%)	28.0	NR
6	Tachiiri et al. Cancers 2024	LEN‐TACE combination therapy (Short‐Term lenvatinib)	Lenvatinib was scheduled to be administered 4 days prior to the first TACE	25	A–B	—	—	Single arm	RECICL	CR, PFS	—	19 (75%)	NR 6‐month PFS rate:81.2% 12‐month PFS rate:75.0%	—
7	Peng et al. J Clin Oncol 2022	LEN plus on‐demand TACE	Patients with treatment‐naive or initially recurrent advanced HCC after surgery were randomly assigned to receive either LEN plus on‐demand TACE (LEN‐TACE) or LEN monotherapy	170	B–C	—	—	Lenvatinib	RECIST 1.1 mRICIST	OS (primary)	78 (45.9%) 92 (54.1%)	1 (0.6%) 5 (2.9%)	10.6	17.8

Notably, our findings represent the first demonstration of the efficacy of this approach in a comparative study. The LEN‐TACE group demonstrated superior radiological response, particularly in terms of CR and ORR (Table 2). Furthermore, the OS and PFS were also superior in the LEN‐TACE group (Figure 2) compared to lenvatinib monotherapy. Moreover, while previous research on LEN‐TACE sequential therapy and combination therapy included patients with BCLC stages A–C, or BCLC‐B cases largely within the up‐to‐7 criteria, our study is distinct in focusing exclusively on patients with BCLC‐B who were deemed unsuitable for TACE.

In summary, our findings indicate that scheduled upfront lenvatinib prior to TACE followed by continuous lenvatinib and on‐demand TACE provides a better prognosis than lenvatinib monotherapy in patients with BCLC‐B, particularly among those unsuitable for TACE, including patients beyond the up‐to‐7 criteria, with infiltrative HCC, or with multiple asynchronous recurrent HCC.

The limitation of this study is its retrospective nature, which introduces an inherent bias. Because this cohort included only patients who actually received lenvatinib, the number of initially screened cases could not be fully determined, which may have caused selection bias. In addition, detailed data on lenvatinib dose intensity were not available, so the impact of dose modifications could not be fully assessed. Furthermore, variability in imaging intervals and missing clinical data across centers may also have influenced outcome assessment. To strengthen our findings, there is a need for future research with prospective external validation. In addition, prospective randomized controlled trials will be needed to further confirm the clinical benefit of this treatment strategy.

Our findings suggest that a scheduled upfront lenvatinib combined with TACE regimen could be particularly advantageous for tumors predicted to be resistant to TACE alone, demonstrating greater therapeutic benefit than lenvatinib monotherapy.

## Author Contributions

**Nobuhito Taniki:** conceptualization (lead), data curation (lead), formal analysis (lead), investigation (lead), methodology (lead), project administration (supporting), resources (equal), visualization (lead), writing – original draft (lead), writing – review and editing (lead). **Keisuke Ojiro:** conceptualization (supporting), data curation (supporting), formal analysis (supporting), resources (equal). **Ryosuke Kasuga:** resources (equal). **Yukie Nakadai:** resources (supporting). **Takaya Tabuchi:** resources (supporting). **Po‐Sung Chu:** supervision (supporting). **Shingo Usui:** supervision (supporting). **Hitomi Hoshi:** resources (equal). **Fumihiko Kaneko:** supervision (supporting). **Akihiro Yamaguchi:** resources (supporting). **Jun Koizumi:** resources (supporting), supervision (supporting). **Hirotoshi Ebinuma:** supervision (supporting). **Masashi Tamura:** resources (supporting), supervision (supporting). **Jitsuro Tsukada:** supervision (supporting). **Masanori Inoue:** supervision (supporting). **Seishi Nakatsuka:** supervision (supporting). **Yasushi Hasegawa:** supervision (supporting). **Yuta Abe:** resources (equal), supervision (supporting). **Minoru Kitago:** supervision (supporting). **Masahiro Jinzaki:** supervision (supporting). **Yuko Kitagawa:** supervision (supporting). **Takanori Kanai:** funding acquisition (lead), project administration (supporting), supervision (supporting). **Nobuhiro Nakamoto:** conceptualization (supporting), project administration (lead), supervision (lead).

## Funding

The authors have nothing to report.

## Ethics Statement

The study protocol was approved by the Keio University School of Medicine Research Ethics Committee, approval number (No. 20160227). The study was conducted in accordance with the principles of the 1975 Declaration of Helsinki and Ethical Guidelines for Medical and Health Research involving Human Subjects (Ministry of Education, Culture, Sports, Science, and Technology and Ministry of Health, Labour and Welfare; Japan). Because this was a retrospective observational study, written informed consent was waived, and an opt‐out consent process was implemented in accordance with institutional guidelines.

## Conflicts of Interest

The authors declare no conflicts of interest.

## Supporting information


**Figure S1:** Treatment protocol of LEN‐TACE.

## Data Availability

Research data are not publicly available on legal or ethical grounds. Further inquiries can be directed to the corresponding author.
