# Supplementary material for: Advantage of Scheduled Upfront Lenvatinib Administration Followed by Transarterial Chemoembolization Therapy Over Lenvatinib Monotherapy in Patients With Unresectable Intermediate‐Stage Hepatocellular Carcinoma: A Multicenter Cohort Study
Source: Cancer Med. 2026 Jan 16;15(1):e71503. doi: 10.1002/cam4.71503 (PMC12811626; doi:10.1002/cam4.71503)
Supplement: Supplementary file 1 — Figure S1: Treatment protocol of LEN‐TACE. [file CAM4-15-e71503-s001.pdf]

Inclusion Criteria

- Unresectable BCLC B HCC
- Meeting any of the following three criteria:
  - Beyond up-to-7 criteria
  - Infiltrative appearance
  - Multiple recurrences (more than 3 times)

Exclusion Criteria

- 20 or more tumors
- ECOG PS 2-4

LEN-TACE ptrotocol

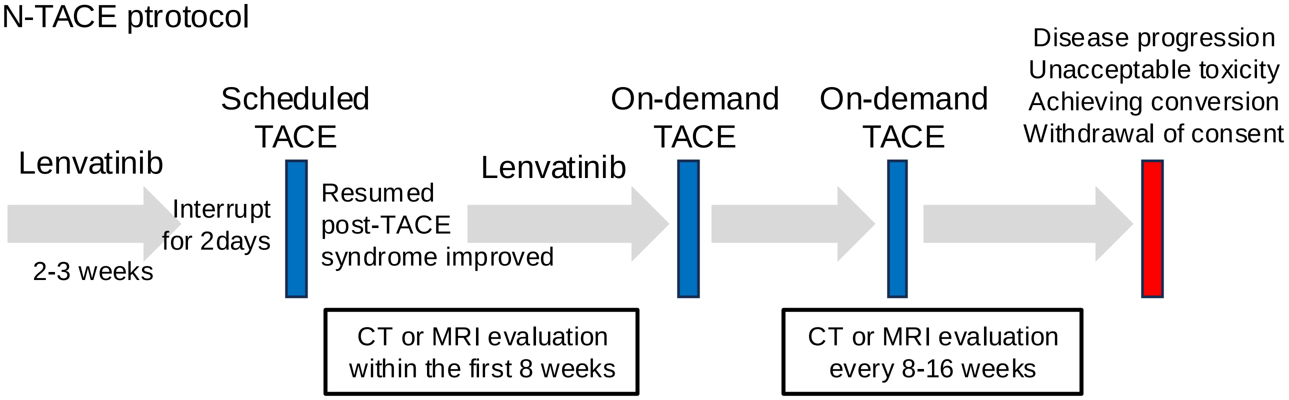

Supplementary Figure 1. Treatment protocol of LEN-TACE
